# A model of yeast glycolysis based on a consistent kinetic characterisation of all its enzymes

**DOI:** 10.1016/j.febslet.2013.06.043

**Published:** 2013-09-02

**Authors:** Kieran Smallbone, Hanan L. Messiha, Kathleen M. Carroll, Catherine L. Winder, Naglis Malys, Warwick B. Dunn, Ettore Murabito, Neil Swainston, Joseph O. Dada, Farid Khan, Pınar Pir, Evangelos Simeonidis, Irena Spasić, Jill Wishart, Dieter Weichart, Neil W. Hayes, Daniel Jameson, David S. Broomhead, Stephen G. Oliver, Simon J. Gaskell, John E.G. McCarthy, Norman W. Paton, Hans V. Westerhoff, Douglas B. Kell, Pedro Mendes

**Affiliations:** a Manchester Centre for Integrative Systems Biology, Manchester Institute of Biotechnology, The University of Manchester, UK; b School of Computer Science, The University of Manchester, UK; c School of Mathematics, The University of Manchester, UK; d School of Chemistry, The University of Manchester, UK; e Faculty of Life Sciences, The University of Manchester, UK; f Centre for Advanced Discovery and Experimental Therapeutics (CADET), Central Manchester University Hospitals NHS Foundation Trust, Manchester Academic Health Sciences Centre, UK; g School of Life Sciences, The University of Warwick, UK; h School of Chemical Engineering and Analytical Science, The University of Manchester, UK; i Cambridge Systems Biology Centre & Department of Biochemistry, University of Cambridge, UK; j Luxembourg Centre for Systems Biomedicine, University of Luxembourg, Luxembourg; k Institute for Systems Biology, Seattle, Washington, USA; l School of Computer Science & Informatics, Cardiff University, UK; m School of Dentistry, The University of Manchester, UK; n Research and Knowledge Transfer, University of Exeter, UK; o Faculty of Medical and Human Sciences, The University of Manchester, UK; p Queen Mary, University of London, UK; q Department of Molecular Cell Physiology, Vrije Universiteit Amsterdam, The Netherlands; r Virginia Bioinformatics Institute, Virginia Tech, USA; s Babraham Institute, Babraham Research Campus, UK

**Keywords:** Glycolysis, Systems biology, Enzyme kinetic, Isoenzyme, Modelling

## Abstract

We present an experimental and computational pipeline for the generation of kinetic models of metabolism, and demonstrate its application to glycolysis in *Saccharomyces cerevisiae*. Starting from an approximate mathematical model, we employ a “cycle of knowledge” strategy, identifying the steps with most control over flux. Kinetic parameters of the individual isoenzymes within these steps are measured experimentally under a standardised set of conditions. Experimental strategies are applied to establish a set of in vivo concentrations for isoenzymes and metabolites. The data are integrated into a mathematical model that is used to predict a new set of metabolite concentrations and reevaluate the control properties of the system. This bottom-up modelling study reveals that control over the metabolic network most directly involved in yeast glycolysis is more widely distributed than previously thought.

## 1 Introduction

A major goal of systems biology is the development of mathematical models of biological phenomena that predict their behaviour accurately, that are able to provide a quantitative explanation of their mechanisms, and that have predictive power as to the effects of changes in their parameters [1–4]. For cellular phenomena, this requires models that represent the action of multiple enzymes as contributors to the behaviour observed. Fortunately, this area of systems biology is built on the strong foundations of enzymology dating from the work of Michaelis and Menten [5], with their model of the dependency of the rate of enzymatic reactions on the concentration of their substrates. Their famous rate law, and similar ones for reversible reactions and reactions with multiple substrates and products, are the building blocks for kinetic models of metabolism.

So far, the majority of mathematical models of biochemical pathways have been developed based on data collected from different sources [6]. The enzyme kinetic data used in such models have usually been measured at each enzyme’s optimal pH; however the optimal pH of each enzyme in a pathway is normally different and the physiological pH and other conditions would not match the optimal conditions for each enzyme [7]. Due to the shortage of comprehensive experimental datasets acquired under specific experimental conditions, this approach may result in a distorted view, i.e., one not fully representative of the system under study. Any unknown parameters of the model are estimated through the finding of a best fit to an available set of experimental data. However, there is potentially an enormous number of parameter values providing the same degree of fitting (whatever the fitting metric), and hence the fitted model may not be a good representation of the real system and may thereby fail to predict its behaviour under different conditions. From a computational perspective this issue has been addressed through sampling approaches [8]. From an experimental perspective the optimal experimental strategy must aim to deliver as comprehensive an information content as possible under a unified set of experimental conditions that should mimic the intracellular environment as much as possible.

In kinetic modelling, the potential for alternative isoenzymes catalysing each metabolic reaction step is generally overlooked. For prokaryotic systems this is likely to be relatively unimportant since a given activity is more commonly controlled by a single gene/protein. However, in eukaryotes, there are often multiple gene/protein isoforms that may act separately and with different activities under different environmental conditions, or may occur in different cellular compartments. For example, Gu et al. [9] have estimated that there are 530, 674, and 1219 duplicate protein families in *Saccharomyces cerevisiae*, *Drosophila melanogaster* and *Caenorhabditis elegans*, respectively. These isoenzymes may play an especially important role in *S. cerevisiae* whose metabolism has evolved through whole-genome duplication and subsequent gene loss [10,11] or divergence [12]. Representation of the distinct isoenzyme activities has not been addressed in any significant kinetic models of metabolic processes to date. Thus, enzymes with identical or similar activities have mostly been integrated together (as in the model of glycolysis of Teusink et al. [13]) and measured as a lumped activity without distinguishing and appreciating the individual characteristics of each isoform. In practice, we can expect differential activities of individual isoenzymes based on changes in the environmental conditions or growth cycle.

In this paper, we introduce a novel strategy that addresses the above sources of heterogeneity by producing complete sets of experimentally-derived data using the same parental strain of *S. cerevisiae* and identical growth conditions. Furthermore, we characterise individual isoforms of enzymes involved in the biochemistry of central carbon metabolism and their differential roles.

As with any system, parameters are distinguished from variables. In a given metabolic network, the parameters are the concentrations of enzymes and their kinetic properties, while the variables are represented by fluxes and concentrations [14]. One strategy therefore isolates the individual components of a system, measures their properties (parameters) in vitro, and uses knowledge of these to reconstruct the network as a mathematical model. This model can then be (and is) used to describe the time-dependent and steady-state concentrations and fluxes in the network. Separate measurements of those variables allow one to test the precision of the model. The model can also serve to highlight potential sources of error in experimental measurements that can then be re-evaluated (e.g., Ref. [15]).

**Fig. 1:**
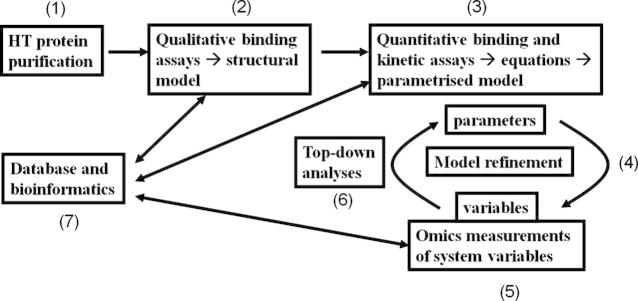
The strategy for bottom-up systems biology used. The meaning of the numbers is described in the text.

Our strategy and workflow [2] for producing and testing systems biology models is summarised in Fig. 1. We have chosen to construct mathematical models of distinct and important areas of metabolism (in terms of the amount of flux carried) and have used *S. cerevisiae* as a model system to develop and validate our approach. We stress a number of features of this approach:

1 The external conditions for the biological system (the cell culture) are well defined, and these act as a source for the metabolites that are taken up by the system.
2 A topological description of the network is then constructed, in which all metabolic reactions are defined and associated to all isoenzymes known to catalyse the reaction. This process is performed by extracting a sub-network from larger, genome-scale metabolic networks, or by compiling literature data [16–20].
3 A kinetic model is built upon the metabolic map defined in point 2. Rate laws and parameter values are either taken from pre-existing models or estimated from published data.
4 The model is evaluated through software packages such as COPASI [21] and used to predict fluxes and metabolite concentrations at steady-state.
5 The control properties of the system are evaluated computationally and the reaction step exerting the major control over the system fluxes is identified.
6 The kinetic properties of the most controlling reaction step are experimentally measured. More particularly, the turnover number (*k_cat_*), concentration and affinity constants of each of the corresponding isoenzymes are measured and their value is used to refine the model.
7 Points 3–5 are repeated until all the reactions in the model have been fully characterised.
8 Once this has been performed for all the elements in the network, the predicted steady-state values of the fluxes and metabolite concentrations are compared with their corresponding experimental measurements.
9 The model’s parameters are then adjusted as appropriate to minimise the mismatch between predicted and measured quantities.
10 All of the relevant data and models are deposited in suitable databases.

As with any new strategy for approaching biochemical (or other) problems, it is appropriate to calibrate or validate it by applying it to a well-understood system. Glycolysis represents an excellent test-case [13,22–24]. It is a universal metabolic pathway conserved across all three domains of life (Archaea, Bacteria and Eukarya, though with some variations) consuming glucose to produce free energy in the form of ATP and reducing equivalents in the form of NADH, and precursors that are used in other cellular metabolic processes. Glycolysis is also of applied interest for the purposes of biomass production in the baking industry [25], as a catalyst for a variety of biotransformations [26–28] and in ethanol production [29–30].

*S. cerevisiae* is also a particularly suitable organism in which to perform such a study from a number of other perspectives: it is well-characterised genetically, and it can be grown under conditions of continuous culture in which its metabolism can adopt a steady state [31–33]. These conditions allow multiple proteome and metabolome samples to be acquired, and ensure a high reproducibility of measurements and hence fidelity of the final results.

This article describes the overall strategy set out above and illustrates the accuracy of systems biology modelling that is currently achievable with such a strategy. This is also the first time that the individual contributions by different isoenzymes have been accounted for in such detail, whether in a model or experimentally.

## 2 Materials and methods

### 2.1 Enzyme production and purification

Enzymes were expressed in *S. cerevisiae* strains that contain either an overexpression plasmid with the open reading frame of interest, which was under control of the *GAL1* inducible promoter and in fusion to a multiple tag at the C-terminus (Yeast ORF Collection, [34]) and N-terminus (Yeast GST-Tagged Collection [35]) or a chromosomally integrated gene with a tag fusion (TAP collection [36]). All collections are available from Open Biosystems (http://www.thermoscientificbio.com/openbiosystems/). The expressions and purification of proteins was performed as described by Malys et al. [37–38]. The success of each step of purification for each protein was assessed by analysing samples from the intermediate and final protein preparations using SDS–PAGE. The amount and concentration of the purified enzyme was determined using QuantiPro™ BCA Assay Kit (Sigma–Aldrich) according to the manufacturer’s recommendations. The quality of the enzyme preparation was further assessed using a 2100 Bioanalyzer (Agilent Technologies).

Although the overexpression of proteins with tags provides significant advantages by increasing throughput and protein recovery from the cell extract, it also brings some limitations related to the function and structure of enzymes. The position of the tag on the protein may interfere with its folding, multimeric complex formation, or functionality. Therefore, in some cases, as for example, enolase 2 (ENO2, EC 4.2.1.11) an alternative construct with an N-terminal tag had to be used. The use of Yeast MORF and Yeast GST-Tagged collections [34–35] in combination allows the purification of 80% of all *S. cerevisiae* proteins.

Applying a standardised approach of using a single yeast expression collection did not allow the reconstitution of fully active phosphofructokinase (EC 2.7.1.11) from individual Pfk1 and Pfk2 subunits. Therefore, in the second round of the experimental cycle, *PFK1* and *PFK2* were co-expressed and their protein products co-purified as a heterooctamer from the Yeast TAP-Tagged collection. The activity of this complex was significantly higher than that of each isoenzyme purified alone.

### 2.2 Enzyme kinetic assays

To determine the kinetic parameters for individual enzymatic reactions, spectrophotometric assays were performed for the glycolytic isoenzymes measuring the consumption or production of NADH or NADPH by using one or more coupling reactions when needed. All assays were carried out in medium-throughput measurements with a BMG Labtech NOVOstar plate reader (automated fluorescence/FP/absorbance reader, Offenburg, Germany) in 384-well format plates with a 60 μl reaction volume [39]. All assays were performed in a standardised reaction buffer at 30 °C and were automated so that all reagents in the reaction buffer (including any coupling enzymes) are in 45 μl, the enzyme (to be assayed) in 5 μl and the substrate in 10 μl volumes. For each individual enzyme, the forward and the reverse reaction were assayed whenever possible. Assays for each individual enzyme were either developed or modified from previously published methodology to be compatible with the conditions of the assay reactions (e.g. pH compatibility or unavailability of commercial substrates).

All assays were coupled with enzyme(s) in which NAD(P) or NAD(P)H is a product or substrate so that its formation or consumption could be followed spectrophotometrically at 340 nm using an extinction coefficient of 6.62 mM^−1^ cm^−1^, unless the reaction of a particular enzyme consumes or produces NADH or NADPH in which case no coupling enzymes were used. Some assays were modified by altering the concentration of coupling enzymes or other reagents to ensure that the rate measured is the rate of the reaction of interest (fully rate limiting). This is a critical step that needs special care to be taken when using coupling enzymes in assays [40].

All measurements are based on at least duplicate determination of the reaction rates at each substrate concentration. Control experiments were run in parallel to check and correct for any background activity. For each isoenzyme, the initial rates at various substrate concentrations were determined and the data obtained were analyzed by the KineticsWizard [41], fitting to Michaelis–Menten kinetics:

$$v=E\;k_{\mathrm{cat}}S/(K_{m}+S)\text{.}$$

Not all enzymes were found to exhibit these kinetics. As reported previously [42], triose phosphate isomerase (TPI1, EC 5.3.1.1) showed substrate inhibition in the direction of dihydroxyacetone phosphate production and was analysed using COPASI [21] according to the following rate law:

$$V=E\;k_{\mathrm{cat}}S/(K_{m}+S(1+{\left(S/K_{i}\right)}^{4}))\text{.}$$

### 2.3 Continuous cultures of *S. cerevisiae*

Continuous cultures of *S. cerevisiae* strain Y23925 (a heterozygous deletion derivative of the diploid BY4743: *MAT**a**/MATα*; *his3*Δ1/*his3*Δ1; *ho*::*Kan*MX4/*HO*; *leu2*Δ0/*leu2*Δ0; *LYS2*/*lys2*Δ0; *met15*Δ0/*MET15*; *ura3*Δ0/*ura3*Δ0) were established in a turbidostat-like approach in which the biomass was monitored by measuring the electrical capacitance of the culture [43–46]. A feedback loop was applied to regulate the addition of growth medium such that the biomass was maintained at 75% of the maximum achieved biomass yield for that medium, where a steady state was obtained (cf. [47]). The growth of the cultures was thus performed without nutrient limitation and at μmax. Samples for quantification of both protein levels and intracellular metabolite concentrations were collected from these continuous cultures as described below.

### 2.4 Proteomics

*S. cerevisiae* cells from 50 ml cultures were harvested by centrifugation for 5 min at 4000 g, and the cells mechanically disrupted using a mini bead-beater (Biospec Products Inc., Bartlesville, USA; http://www.biospec.com/) yielding the cellular cytoplasmic soluble fraction for analysis. The latter was combined with known amounts of the recombinant labelled QconCAT protein (containing diagnostic peptides for the glycolytic enzymes [48]), and co-digested to completion with trypsin. The resulting peptides were diluted and resolved over a linear incrementing solvent gradient by LC-MS using a nanoACQUITY chromatograph (Waters MS Technologies) coupled to an LTQ-Orbitrap XL (ThermoFisher Scientific). Automated data analysis and subsequent calculations were carried out using the QconCAT PrideWizard [49].

### 2.5 Metabolomics

For metabolomics, a quenching method was applied [50] which sampled 10 ml of culture solution into 40 ml of a 60:40 methanol/water solution stored at a temperature of −47 °C [51]. Immediate separation of cells was performed by applying centrifugation (4000 g for 5 min) followed by removal of the quenching solution. Both were stored at −80 °C prior to extraction or analysis. The intracellular metabolite pool was extracted into 1250 μl of an 80:20 water/methanol solution with cell wall disruption provided by eight freeze–thaw cycles to ensure full metabolite recovery. Samples were analysed using two analytical platforms. Fructose-1,6-bisphosphate was quantified applying UPLC-MS (Waters Acquity UPLC coupled to a ThermoFisher hybrid electrospray LTQ-Orbitrap mass spectrometer (ThermoFisher Scientific, http://www.thermofisher.com/)) and the standard addition method for accurate quantification. Other metabolites were quantified applying GC–MS (Agilent 6890 Gas Chromatograph coupled to a Leco Pegasus III electron impact-time-of-flight mass spectrometer (Leco, http://www.leco.com/)) and the external calibration method for accurate quantification. The concentrations determined in the extracted samples were reported as the number of molecules per cell.

The gas chromatographic separation of 2-phosphoglycerate and 3-phosphoglycerate is technically demanding and was not achieved during this study. Therefore, the combined concentration of both metabolites was reported rather than separate concentrations for each metabolite.

### 2.6 Modelling

An existing model of yeast glycolysis [23] was modified so that any fixed (‘clamped’) fluxes in that model were redefined as first-order in their reactants. Where possible, initial metabolite concentrations were updated according to in-house values; otherwise recent literature data for respiro-fermentative, non-starved cells were used [24].

At each of 17 iterations, flux control coefficients [52–53] were calculated using COPASI [21]. Where possible, the uncharacterised reaction with the highest control over glucose uptake was then replaced with kinetic and proteomic data for each isoenzyme; otherwise literature data were used (Table 1). HXT parameters were taken from van Eunen et al. [24] (iteration 1); a saturative generic ATPase rate law (representing the collective of all ATP consuming processes) was taken from Teusink et al. (1998) [54] (iteration 3); the glycerol branch was replaced with the two-reaction model of Cronwright et al. [55] (iteration 4); the glycogen and trehalose branches were replaced with the five-reaction model of Smallbone et al. [56] (iteration 6); acetate and succinate branches were taken from van Eunen et al., 2012 (iteration 7); TPI function and parameters were taken from Mendes et al. [42], though these were measured in-house (iteration 17). We also modified the kinetics of PFK to take account of its thermodynamic reversibility. Subsequent fitting was performed in COPASI.

All models are provided in standard Systems Biology Markup Language format (SBML level 2 version 4; [57]) embedded with principled and computer-readable annotations [17,58–59]. The models are available from the BioModels database [60] and in the JWS Online [61] , with the accession numbers given in Table 1.

### 2.7 Informatics infrastructure

The production of this model was supported by the development of an informatics infrastructure to aid with capture and analysis of experimental data. The toolset includes support for enzyme kinetics [41], quantitative proteomics [49], and quantitative metabolomics data [62–63]. Furthermore, workflows have been generated to automate the generation and parameterisation of kinetic models [64–66].

## 3 Results and discussion

### 3.1 A standardised pipeline from network to kinetic model

In order to describe a biological system quantitatively, the characteristics of all its components need to be established. Where possible, they should represent a system in steady state, where all measurements, even when done at different times, are performed under identical conditions. Therefore, a controlled turbidostat culture of *S. cerevisiae* strain YDL227C was grown under strictly defined conditions as described in Section 2, from which cell extracts were prepared for quantifying protein and metabolite concentrations. This pipeline approach was driven by the aim of using robust and standardised experimental strategies that can easily be transferred to the characterisation of other biochemical networks. It involved three distinct experimental platforms: enzyme kinetics, quantitative metabolomics and quantitative proteomics.

In order to characterise the kinetics of isoenzymes, proteins were expressed and purified from commercially available yeast strain collections. Kinetic constants for these enzymes weredetermined as described in Materials and Methods, leading to the set of parameters given in Table 2. The same table also compares these with some previously published values, although those measurements were taken from a different strain and at a different pH and under a variety of other conditions.

To establish the absolute quantities of these individual isoenzymes in the cell, the complete protein content was extracted from turbidostatic yeast cultures and quantified using the QconCAT approach, according to methods described previously [48,67] and in Section 2. These concentrations are listed in Table 3.

The complete metabolite contents were extracted from the quenched yeast cells of the same turbidostat culture and quantitatively measured by Ultra Performance Liquid Chromatography (UPLC-MS [68]) and Gas Chromatography-Mass Spectrometry (GC–MS [69]) using standard solutions of known concentration as described in Section 2. A summary of the metabolite concentrations so determined is shown in Table 4.

From a modelling perspective, the enzyme kinetic constants and protein concentrations represent the parameters of the system, and the metabolite concentrations represent the variables. Combining the protein concentration data with those for the enzyme kinetic parameters, together with the measured steady state metabolite levels, allows a mathematical model of yeast glycolysis to be produced for this system, which is provided in standard SBML [57]. A major difference in the reactions of our model when compared to previous efforts in modelling metabolism (such as [13,23]) is that isoenzymes are represented explicitly. In addition, the enzyme kinetic parameters have been measured under standard conditions designed to mimic to a greater or lesser extent the intracellular environment.

### 3.2 The cycle of knowledge is crucial for systems biology

Scientific advance may be seen as an iterative cycle linking knowledge and ideas to observations and data [2,70–72]. Deductive reasoning uses background knowledge to construct (by unstated means) a hypothesis that is tested experimentally to produce observations. By contrast, inductive (or abductive) reasoning is purely data-driven, generating more general (or specific) hypotheses, rather than starting with them. Because of the high dimensionality of typical data, computer-intensive methods are required to turn the data into knowledge.

This inductive–deductive “cycle of knowledge” is crucial for systems biology: predictive mathematical models are used to interpret, organise and integrate the range of available experimental data. The models produce hypotheses about the underlying complex system dynamics, which are tested experimentally. The models are then more closely validated and updated, continuing the cycle.

Many turns of the cycle of knowledge were required to produce this model. An existing model [23] was found not to predict our measured metabolite levels adequately (Table 4). Thus at each iteration, the most important reaction to characterise was identified through its control over glucose uptake. This ‘most important’ reaction was then characterised experimentally, or through literature data.

For some iterations, the addition of experimental data led to a significant decrease in both fit to our data and flux through the pathway. This was because the limiting rate (*V*) of these reactions – a function of both protein concentration and turnover number – was now too low (i.e. was lower than the estimated in vivo flux). By contrast, using all expression data simultaneously provides substantial constraints in which non-systematic noise cancels out effectively [73]. In vitro and in vivo enzymatic activities may differ for a number of reasons such as missing or unknown effectors, cellular crowding or channelling. However, they may also differ due to inactivation of the enzyme in the purification or assay methods, or by the presence of the purification tag at the N- or C-terminus. By applying alternative purification and assay techniques to isoenzymes with low activity, new data were collected, allowing the model to be refined and improving its consistency with the observations. Four cycles of experiment and modelling were thus carried out.

For phosphofructokinase a *k_cat_* value of 3 s^−1^ was first determined, which was well below what should be expected to match the actual glycolytic flux. However this is because the α and β subunits (products of different genes, *PFK1* and *PFK2*) were purified and assayed in isolation. A subsequent assay combined the α and β subunits, that had been purified separately, in equimolar amounts, but this still produced a low *k_cat_* value. Finally the two subunits were co-expressed, co-purified and then assayed, resulting in a *k_cat_* value of 200 s^−1^.

The reaction catalysed by the enolases was also deficient in total capacity relative to the observed physiological flux: the *k_cat_* of Eno1p was 0.8 s^−1^ and Eno2p was inactive. Since these enzymes had been previously purified and stored at −70 °C, the new experiment assayed them immediately after purification. In this case Eno1p had a *k_cat_* of 8 s^−1^ but Eno2p was still inactive, and the activity of Eno1p alone was not enough to explain the flux. A hypothesis was formulated that the tag in the C-terminus of Eno2p (which is left on in the MORPH strain proteins) could be interfering with its active site. Published evidence for the role of the C-terminus of enolase in enzyme activity and in binding Mg^2+^ were found [74–75]. Thus a new construct was obtained for *ENO2* with a tag in the N-terminus, it was expressed, purified and assayed fresh, resulting in a *k_cat_* of 20 s^−1^, which is more than twice as active as Eno1p.

The calculated limiting rate of the reaction catalysed by the pyruvate decarboxylases, EC 4.1.1.1, was also below the physiological flux. The *k_cat_* values determined were: Pdc1p 3 s^−1^, Pdc5p 2 s^−1^, and Pdc6p was inactive. These enzymes had been stored at −70 °C following purification. We re-purified them and performed the assay immediately after the purification. This time, the *k_cat_* values for the three enzymes were all 10 s^−1^, which leads to the conclusion that the storage had caused loss of activity.

The enzyme concentration of fructose bisphosphate aldolase (Fba1p, EC 4.1.2.13) was updated from 5 × 10^5^–4 × 10^6^ molecules per cell, after reinterpretation of the proteomics data. Lysine acetylation occurred on an internal lysine residue of an analyte peptide from Fba1p. This led to division of the analyte signal between the modified and unmodified forms, present in our data, and resulted in an underestimation of the total analyte signal [48]. Thus the inclusion of post-translational modifications as well as isozyme data can lead to more precise estimations of relevant kinetic parameters.

It is important to note that experimental problems such as those described above only came to light upon modelling of the entire system. Since this project included iterative modelling and experimental components, replicate experiments could be carried out when needed. This contrasts with previous modelling approaches that typically relied upon third-party experimental data. It also highlights that the iterative and integrative modelling approach followed here has an important role in quality control for the experimental data, which would not be applicable to dedicated experimental studies that do not contain a modelling element.

**Fig. 2:**
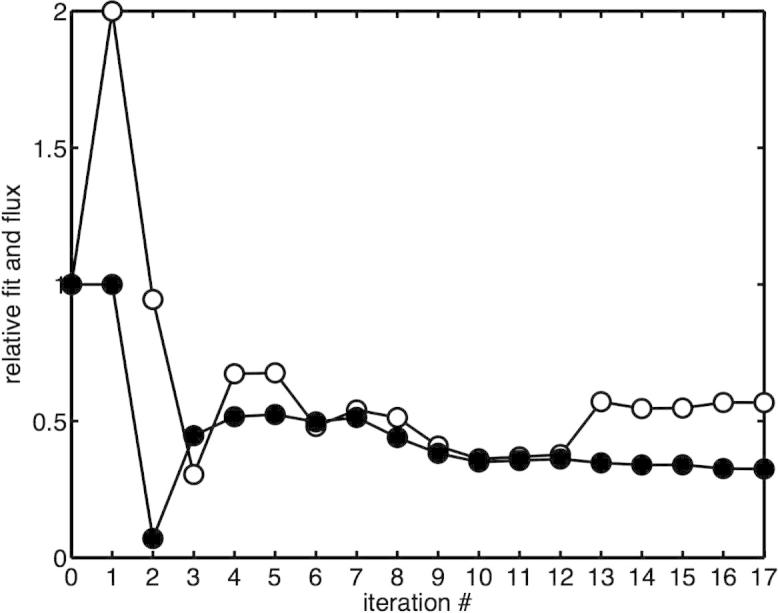
Improvement in fit between model predictions and data as more reactions are characterised. The filled circles denote the fit as determined by normalised root mean square difference between the measured and experimental metabolite concentrations. Also shown is the glucose uptake rate (open circles). Both fit and flux are rescaled to take value unity for the initial model.

In Fig. 2, we present the change in the fit between model predictions and measured metabolite concentrationsthrough each iterative characterisation. While an improvement in fit between the initial model and that fed with our parameter values is seen, there is little change in fit or flux after five iterations. As such, it is vital for model predictivity only to characterise those reactions with the highest control under standard conditions; for reactions with lower control, other sources may be used.

### 3.3 Effective cytoplasmic volume is an important parameter

Whilst many parameters were measured under standardised conditions, some were nonetheless estimated from the literature. The relative contribution of each unmeasured parameter to the quality of fit to data may be ranked using sensitivity analysis (see e.g., Ref. [76]). We defined a number of fitting criteria: sufficient closeness of predicted to observed metabolite concentrations, no drop in flux into glycolysis, and an energy charge of at least 0.8 [77]. If we were unable to satisfy these criteria by fitting only the most important parameter, we tried using two parameters, and continued the cycle until complete.

The most important parameter was found to be the cytoplasmic volume, whose role is to convert metabolite and protein copy number to molar concentration (the units in which the Michaelis–Menten parameters are measured). Given estimates of cell volume of 40 fl [78–79], of which around 50% is cytoplasm [80], we initially estimated the cytoplasm to be 20 fl. However, following fitting, we found that a volume of 5 fl provided a much better fit to our data. This could be explained by macromolecular crowding [81], which has the effect that the space available for water and dissolved molecules is much smaller than the total space, and so the effective volume is lower. It is also important to realise that our model represents a bulk average [82–83], so the volume should reflect the average volume of cells. Actively growing cultures are dominated by new cells recently formed by budding that have not undergone division – these cells are smaller than those that have already divided. Further, older cells have large vacuoles that occupy most of the cellular space and their cytoplasmic volume is considerably smaller than the total cellular volume [84].

The other two changes in parameter values required were both related to bulked reactions: the ATPase rate was increased (*V* from 1.1 mM s^−1^ to 5.3 mM s^−1^) and the succinate branch was turned off (*k* from 0.005 s^−1^ to 0).

### 3.4 Is 3-phosphoglycerate a substrate for enolase 1?

The assays performed for phosphoglycerate mutase (GPM, EC 5.4.2.1) in the direction of 3-phosphoglycerate (P3G) to 2-phosphoglycerate (P2G) revealed an interaction between the substrate P3G and the coupling enzymes system (specifically the enolase). This made it difficult to determine the kinetic properties of GPM for the forward reaction, and hence only the kinetics for the reverse reaction could be determined.

In studying this interaction, it was found that P3G acts as a substrate for Eno1p (*k_cat_* = 5.07 s^−1^ ± 2.9% and *K_m_ *= 0.617 mM ± 10.2%), producing phophenolpyruvate (PEP). Multiple batches from different sources were tested and ^31^P NMR spectra of the P3G material showed no evidence for the presence of P2G, hence this observation is not due to P2G contamination. There is some evidence [85] that ENO and GPM form a metabolic channel [86]; since our model assumes no channelling, this was not incorporated, but is noted here as a discovery of interest.

### 3.5 Separating kinetic parameters from protein expression levels reveals individuality of isoenzymes

In this work, we constructed a model based on three data sources: metabolomics, proteomics and enzyme kinetics. In contrast to earlier work, we separated the limiting rates into two terms representing (the product of) the enzyme level and its maximal turnover number: *V_max_* = [*E*]⋅*k_cat_*. This is important because the in vivo limiting rate is near impossible to determine. Specifically, it is not possible to deconvolve the activity of different isoenzymes from cell-free extracts measurements. The strategy we followed is to determine separately [*E*] (by quantitative proteomics) and *k_cat_* (using enzyme kinetics). Here the default assumption is that the *k_cat_* of enzymes is not changed by the processes of protein purification; since we purify proteins that have tags in their C-terminus, we also assume that the *k_cat_* (and other kinetic parameters) are not changed by the presence of the tag. The latter condition is more likely to be violated in some cases, so we checked for this. Indeed, we have found that Eno2p activity was affected by C-terminus tags (see above).

The benefits of this separation are twofold. First, it allows one to investigate the effects of the different isoenzymes within the system. For example glucokinase (Glk1p, EC 2.7.1.2) has lower activity than the two hexokinases (Hxk1p and Hxk2p, EC 2.7.1.1); however it also has a higher affinity for its substrate glucose (see Table 2), allowing it to operate effectively at low substrate concentrations.

Secondly, the kinetic characteristics of enzymes remain fairly constant under different growth conditions. Rather, cells respond to cues through changing enzyme levels, validating the assumptions stated above. As such, knowledge of enzyme levels under different physiological conditions may be combined with a specific set of kinetic data to create a suite of mathematical models of a system.

### 3.6 Metabolic control analysis

In Table 5, we present the flux-control coefficients (FCC [52]) for the initial and final models. For the initial model, the three main controllers of glucose uptake appeared to be glucose transport, hexokinase and ATPase. These same three also exert the most control in the final model, though through decomposition we find that it is specifically Hxk2p (FCC 1.91), rather than Hxk1p (FCC 0.08) or Glk1p (FCC 0.001) that exerts most of the flux control. Overall, we found in the original model that 95% of the total control is exerted by these three reactions plus PFK. By contrast, in the final model, control is distributed more widely over the whole system, with 95% of control distributed over ten reactions. This represents a substantial change in the picture as our understanding was refined. This is also different from distributions in other organisms; for example, in *Trypanosoma brucei*, control over glycolytic flux was found to be almost uniquely determined by the glucose transporter [87]. These results are also in agreement with the view of Kacser and Burns [88] that the control of metabolism is, in general, and necessarily, widely distributed.

## 4 Conclusion

For over a decade, modern systems biology has been seen as a crucial strategy and methodology for biological research. It pays particular attention to the way in which molecules interact inside cells in a quantitative way. Within systems biology there are two fundamentally distinct approaches, one uses data from large-scale measurements (transcriptomics, etc.) to reverse engineer models directly from those data; the other approach is based on combining enzyme kinetic data to build mechanistic biochemical network models. The latter approach, sometimes referred to as “bottom-up”, is a descendent of the work of Michaelis and Menten, and at one level uses essentially the same methodology to assess the properties of each of the component enzymes in the system. It has thus been common to re-use, in systems biology models, the enzyme kinetic parameters previously estimated inenzymology studies (e.g., Ref. [13]). The present work improves on this practice by recognising that those enzymology studies were carried out under different conditions for each enzyme, while in cells the actual conditions are (at least, nominally) common to all enzymes of the pathway residing in a given compartment. Here, all enzymes – and importantly all isoenzymes – were assayed in standard conditions, such that the resulting model is self-consistent and physiologically relevant. The approach taken here followed the cycle of knowledge approach [2,70–72] as several iterations of experiments and modelling were performed. The final model reveals that the control of the pathway is considerably more distributed than was thought in the past based on previous models, and is in agreement with both experimental evidence and theoretical insights [88]. This study provides a clear example of how bottom-up systems biology, based on the solid foundations laid out 100 years ago by Michaelis and Menten [5], is useful in discovering and understanding new biochemistry.

## Figures and Tables

**Table 1:** Sequence of models generated through the cycles of experiment and modelling. At each iteration, the uncharacterised reaction with the highest control over glucose uptake was then replaced with our experimental data or literature data (see text for details). Each version of the model is available from the BioModels database [60]. For example, the final model may be accessed at http://identifiers.org/biomodels.db/MODEL1303260018. The models are also available from the JWS Online [61], where they can be simulated online at http://jjj.mib.ac.uk/database/smallbone.

Iteration	Reaction	BioModels id	JWS Online id
0		MODEL1303260000	Smallbone0
1	HXT	MODEL1303260001	Smallbone1
2	HXK	MODEL1303260002	Smallbone2
3	ATPase	MODEL1303260003	Smallbone3
4	Glycerol_branch	MODEL1303260004	Smallbone4
5	PFK	MODEL1303260005	Smallbone5
6	Glycogen_branch	MODEL1303260006	Smallbone6
7	Succinate_branch	MODEL1303260007	Smallbone7
8	PDC	MODEL1303260008	Smallbone8
9	TDH	MODEL1303260009	Smallbone9
10	FBA	MODEL1303260010	Smallbone10
11	PGI	MODEL1303260011	Smallbone11
12	ENO	MODEL1303260012	Smallbone12
13	PGK	MODEL1303260013	Smallbone13
14	ADH	MODEL1303260014	Smallbone14
15	GPM	MODEL1303260015	Smallbone15
16	PYK	MODEL1303260016	Smallbone16
17	TPI	MODEL1303260017	Smallbone17
18		MODEL1303260018	Smallbone18

**Table 2:** Enzyme kinetics parameters determined in this project and contrasted with those of [23].

Reaction	Isoenzyme	Parameter	Value	SEM (%)	Ref. [23]	Unit
ADH	Adh1p	kcat	176	1.3		s^−1^
ADH	Adh1p	Kacald	0.462	5.3	1.11	mM
ADH	Adh5p	kcat	0	0		s^−1^
ENO	Eno1p	kcat	7.6	1.9		s^−1^
ENO	Eno1p	Kp2g	0.043	10	0.04	mM
ENO	Eno2p	kcat	19.9			s^−1^
ENO	Eno2p	Kp2g	0.104		0.04	mM
FBA	Fba1p	kcat	4.14	1.5		s^−1^
FBA	Fba1p	Kf16bp	0.451	5.3	0.3	mM
GPM	Gpm1p	kcat	400			s^−1^
GPM	Gpm1p	Kp2g	1.41		0.08	mM
HXK	Glk1p	kcat	0.0721			s^−1^
HXK	Glk1p	Kglc	0.0106		0.08	mM
HXK	Glk1p	Katp	0.865		0.15	mM
HXK	Hxk1p	kcat	10.2			s^−1^
HXK	Hxk1p	Kglc	0.15		0.08	mM
HXK	Hxk1p	Katp	0.293		0.15	mM
HXK	Hxk2p	kcat	63.1			s^−1^
HXK	Hxk2p	Kglc	0.2		0.08	mM
HXK	Hxk2p	Katp	0.195		0.15	mM
PDC	Pdc1p	kcat	12.1	3.5		s^−1^
PDC	Pdc1p	Kpyr	8.5	10	4.33	mM
PDC	Pdc5p	kcat	10.3	2.1		s^−1^
PDC	Pdc5p	Kpyr	7.08	5.7	4.33	mM
PDC	Pdc6p	kcat	9.21			s^−1^
PDC	Pdc6p	Kpyr	2.92		4.33	mM
PFK	Pfk1p:Pfk2p	kcat	210	1.8		s^−1^
PGI	Pgi1p	kcat	487	3.7		s^−1^
PGI	Pgi1p	Kg6p	1.03	19	1.4	mM
PGI	Pgi1p	Kgf6p	0.307	6.8	0.3	mM
PGK	Pgk1p	kcat	58.6			s^−1^
PGK	Pgk1p	Kp3g	4.58		0.53	mM
PGK	Pgk1p	Katp	1.99		0.3	mM
PGK	Pgk1p	nHadp	2			
PYK	Cdc19p	kcat	20.1	2.9		s^−1^
PYK	Cdc19p	Kpep	0.281	12	0.14	mM
PYK	Cdc19p	Kadp	0.243	13	0.53	mM
PYK	Pyk2p	kcat	0	0		s^−1^
TDH	Tdh1p	kcat	19.1	1.5		s^−1^
TDH	Tdh1p	Kgap	0.495	7.5	0.21	mM
TDH	Tdh2p	kcat	8.63			s^−1^
TDH	Tdh2p	Kgap	0.77		0.21	mM
TDH	Tdh3p	kcat	18.2	1.8		s^−1^
TDH	Tdh3p	Kgap	0.423	9.2	0.21	mM
TDH	Tdh3p	Kbpg	0.909		0.0098	mM
TPI	Tpi1p	kcat	564	1.5		s^−1^
TPI	Tpi1p	Kdhap	6.45	5		mM
TPI	Tpi1p	Kgap	5.25	12		mM
TPI	Tpi1p	Kigap	35.1	3.1		mM

**Table 3:** Protein concentrations measured by QconCat, expressed as protein molecules per cell. For comparison the data determined in [36] is also displayed.

Reaction	Isoenzyme	Uniprot id	Molecules/cell	SEM (%)	Ref. [36]
ADH	Adh1p	P00330	494 000	1.1	0
ADH	Adh5p	P38113	12 800	8.1	1310
ENO	Eno1p	P00924	2 070 000	0.9	76 700
ENO	Eno2p	P00925	5 950 000	0.7	2610
FBA	Fba1p	P14540	4 030 000		1 020 000
GPM	Gpm1p	P00950	2 200 000	0.6	172 000
HXK	Glk1p	P17709	136 000	5.5	21 100
HXK	Hxk1p	P04806	50 500	1.1	40 800
HXK	Hxk2p	P04807	185 000	0.6	114 000
PDC	Pdc1p	P06169	3 220 000	0.8	8970
PDC	Pdc5p	P16467	37 200	19.1	471 000
PDC	Pdc6p	P26263	19 700	1.7	1520
PFK	Pfk1p	P16861	141 000	0.7	89 800
PFK	Pfk2p	P16862	118 000	2.8	90 200
PGI	Pgi1p	P12709	416 000	0.6	91 600
PGK	Pgk1p	P00560	776 000	1.5	314 000
PYK	Cdc19p	P00549	6 170 000	1.3	291 000
PYK	Pyk2p	P52489	18 300	11.4	2130
TDH	Tdh1p	P00360	1 060 000	1.2	120 000
TDH	Tdh2p	P00358	0		121 000
TDH	Tdh3p	P00359	12 700 000	0.9	169 000
TPI	Tpi1p	P00942	886 000	0.8	207 000

**Table 4:** Measured and predicted metabolite concentrations. Experimental values determined as described in Methods. Concentrations were calculated relative to an effective cytoplasmic volume of 5 fl (see main text). Predictions were calculated with the final model (iteration 18 in Table 1). Data from [23] is presented for comparison.

ID	Name	ChEBI id	Molecules/cell (×10^3^)	SEM (%)	Concentration (mM)		
					Observed	Predicted	Ref. [23]
DHAP	Dihydroxyacetone phosphate	16 108	3500	18.9	1.162	1.584	1.004
F16bP	Fructose 1,6-bisphosphate	28 013	13 800	20.2	4.583	2.780	6.221
F6P	Fructose 6-phosphate	16 084	709	14.8	0.235	0.325	0.625
G3P	Glycerol 3-phosphate	15 978	825	24.3	0.274	0.226	0.150
G6P	Glucose 6-phosphate	17 665	2330	9.7	0.774	1.213	2.675
GAP	Glyceraldehyde 3-phosphate	29 052	951	26.9	0.316	0.067	0.045
GLC	Glucose	4167	18 900	4.6	6.277	0.635	0.098
PEP	Phosphoenol-pyruvate	18 021	1840	23.5	0.611	0.477	0.063
PYR	Pyruvate	15 361	6350	16.6	2.109	3.329	1.815
P2G	Glycerate	17 835	1620	17.7	0.538	0.613	1.013
P3G	Phosphates	17 794					

**Table 5:** Flux-control coefficients on the glucose input flux, for the initial and final models (iterations 0 and 18 in Table 1).

Reaction	Flux-control coefficients	
	Model 0	Model 18
HXT	0.844	1.467
HXK	0.201	1.995
ATPase	−0.076	−3.303
PFK	0.045	0.356
Glycerol_branch	−0.017	
Glycogen_branch	−0.009	
Succinate_branch	−0.008	
TDH	0.007	0.118
ADH	0.006	0.359
Trehalose_branch	−0.006	
FBA	0.005	0.102
PGI	0.005	0.023
ENO	0.003	0.016
PGK	0.000	0.172
PYK	0.000	0.063
GPM	0.000	−0.044
PDC	0.000	0.001
TPI	0.000	0.008
AK	0.000	0.000
