# Supplementary material for: A model of yeast glycolysis based on a consistent kinetic characterisation of all its enzymes
Source: FEBS Lett. 2013 Sep 2;587(17):2832–41. doi: 10.1016/j.febslet.2013.06.043 (PMC3764422; doi:10.1016/j.febslet.2013.06.043)
Supplement: Supplementary data — Supplementary material. [file mmc1.pdf]

Introduction

This document constitutes supplementary material for the article by Smallbone *et al.* (2013) “A model of yeast glycolysis based on a consistent kinetic characterization of all its enzymes”. It contains the full final mathematical model (model 18 in the manuscript). Note, however, that this is a transcription of the electronic version of the model to LaTeXwhich, although checked, may still contain errors. The definitive version of all models in this article are the electronic versions deposited in the BioModels database, with accession numbers MODEL1303260000–MODEL1303260018 (accessible from e.g. http://identifiers.org/biomodels.db/MODEL1303260018).

Rate laws

$$v_{\text{acetate branch}} = k_{(\text{acetate branch})} \cdot [\text{acetaldehyde}] \cdot [\text{NAD}]$$

$$v_{\text{ADH1}} = \frac{[\text{ADH1}] \cdot \text{kcat}_{(\text{ADH1})} \cdot \left(\frac{[\text{acetaldehyde}] \cdot [\text{NADH}]}{\text{Kacald}_{(\text{ADH1})} \cdot \text{Kinadh}_{(\text{ADH1})}} - \frac{[\text{ethanol}] \cdot [\text{NAD}]}{\text{Kacald}_{(\text{ADH1})} \cdot \text{Kinadh}_{(\text{ADH1})} \cdot \text{Keq\_ADH}} \right)}{1 + \frac{[\text{NADH}]}{\text{Kinadh}_{(\text{ADH1})}} + \frac{[\text{acetaldehyde}] \cdot \text{Knadh}_{(\text{ADH1})}}{\text{Kinadh}_{(\text{ADH1})} \cdot \text{Kacald}_{(\text{ADH1})}} + \frac{[\text{ethanol}] \cdot \text{Knad}_{(\text{ADH1})}}{\text{Kinad}_{(\text{ADH1})} \cdot \text{Ketoh}_{(\text{ADH1})}} + \frac{[\text{NAD}]}{\text{Kinad}\ \ mathrm(ADH1)} + \frac{[\text{acetaldehyde}] \cdot [\text{NADH}]}{\text{Kinadh}_{(\text{ADH1})} \cdot \text{Kacald}_{(\text{ADH1})}} + \frac{[\text{NADH}] \cdot [\text{ethanol}] \cdot \text{Knad}_{(\text{ADH1})}}{\text{Kinadh}_{(\text{ADH1})} \cdot \text{Kinad}_{(\text{ADH1})} \cdot \text{Ketoh}_{(\text{ADH1})}} + \frac{[\text{acetaldehyde}] \cdot [\text{NAD}] \cdot \text{Knadh}_{(\text{ADH1})}}{\text{Kinadh}_{(\text{ADH1})} \cdot \text{Kinad}_{(\text{ADH1})} \cdot \text{Kacald}_{(\text{ADH1})}} + \frac{[\text{ethanol}] \cdot [\text{NAD}]}{\text{Ketoh}_{(\text{ADH1})} \cdot \text{Kinad}_{(\text{ADH1})}} + \frac{[\text{acetaldehyde}] \cdot [\text{NADH}] \cdot [\text{ethanol}]}{\text{Kinadh}_{(\text{ADH1})} \cdot \text{Kietoh}_{(\text{ADH1})} \cdot \text{Kacald}_{(\text{ADH1})}} + \frac{[\text{acetaldehyde}] \cdot [\text{ethanol}] \cdot [\text{NAD}]}{\text{Kiacald}_{(\text{ADH1})} \cdot \text{Kinad}_{(\text{ADH1})} \cdot \text{Ketoh}_{(\text{ADH1})}}}$$

$$v_{\text{ADH5}} = \frac{[\text{ADH5}] \cdot \text{kcat}_{(\text{ADH5})} \cdot \left(\frac{[\text{acetaldehyde}] \cdot [\text{NADH}]}{\text{Kacald}_{(\text{ADH5})} \cdot \text{Kinadh}_{(\text{ADH5})}} - \frac{[\text{ethanol}] \cdot [\text{NAD}]}{\text{Kacald}_{(\text{ADH5})} \cdot \text{Kinadh}_{(\text{ADH5})} \cdot \text{Keq\_ADH}} \right)}{1 + \frac{[\text{NADH}]}{\text{Kinadh}_{(\text{ADH5})}} + \frac{[\text{acetaldehyde}] \cdot \text{Knadh}_{(\text{ADH5})}}{\text{Kinadh}_{(\text{ADH5})} \cdot \text{Kacald}_{(\text{ADH5})}} + \frac{[\text{ethanol}] \cdot \text{Knad}_{(\text{ADH5})}}{\text{Kinad}_{(\text{ADH5})} \cdot \text{Ketoh}_{(\text{ADH5})}} + \frac{[\text{NAD}]}{\text{Kinad}\ \ mathrm(ADH5)} + \frac{[\text{acetaldehyde}] \cdot [\text{NADH}]}{\text{Kinadh}_{(\text{ADH5})} \cdot \text{Kacald}_{(\text{ADH5})}} + \frac{[\text{NADH}] \cdot [\text{ethanol}] \cdot \text{Knad}_{(\text{ADH5})}}{\text{Kinadh}_{(\text{ADH5})} \cdot \text{Kinad}_{(\text{ADH5})} \cdot \text{Ketoh}_{(\text{ADH5})}} + \frac{[\text{acetaldehyde}] \cdot [\text{NAD}] \cdot \text{Knadh}_{(\text{ADH5})}}{\text{Kinadh}_{(\text{ADH5})} \cdot \text{Kinad}_{(\text{ADH5})} \cdot \text{Kacald}_{(\text{ADH5})}} + \frac{[\text{ethanol}] \cdot [\text{NAD}]}{\text{Ketoh}_{(\text{ADH5})} \cdot \text{Kinad}_{(\text{ADH5})}} + \frac{[\text{acetaldehyde}] \cdot [\text{NADH}] \cdot [\text{ethanol}]}{\text{Kinadh}_{(\text{ADH5})} \cdot \text{Kietoh}_{(\text{ADH5})} \cdot \text{Kacald}_{(\text{ADH5})}} + \frac{[\text{acetaldehyde}] \cdot [\text{ethanol}] \cdot [\text{NAD}]}{\text{Kiacald}_{(\text{ADH5})} \cdot \text{Kinad}_{(\text{ADH5})} \cdot \text{Ketoh}_{(\text{ADH5})}}}$$

$$v_{\text{AK}} = k_{(\text{AK})} \cdot \left([\text{ADP}] \cdot [\text{ADP}] - \frac{[\text{AMP}] \cdot [\text{ATP}]}{\text{Keq}_{(\text{AK})}} \right)$$

$$v_{\text{ATPase}} = \frac{\frac{\text{Vmax}_{(\text{ATPase})} \cdot [\text{ATP}]}{\text{Katp}_{(\text{ATPase})}}}{1 + \frac{[\text{ATP}]}{\text{Katp}_{(\text{ATPase})}}}$$

$$v_{\text{CDC19}} = \frac{\frac{[\text{CDC19}] \cdot \text{kcat}_{(\text{CDC19})} \cdot \left([\text{phosphoenolpyruvate}] \cdot [\text{ADP}] - \frac{[\text{pyruvate}] \cdot [\text{ATP}]}{\text{Keq\_PYK}} \right)}{\text{Kpep}_{(\text{CDC19})} \cdot \text{Kadp}_{(\text{CDC19})}}}{\left(1 + \frac{[\text{phosphoenolpyruvate}]}{\text{Kpep}_{(\text{CDC19})}} + \frac{[\text{pyruvate}]}{\text{Kpyr}_{(\text{CDC19})}} + \text{L0}_{(\text{CDC19})} \cdot \frac{\frac{[\text{ATP}]}{\text{Kiatp}_{(\text{CDC19})}} + 1}{\frac{[\text{fructose 1,6-bisphosphate}]}{\text{Kf16p}_{(\text{CDC19})}} + 1} \right) \cdot \left(1 + \frac{[\text{ADP}]}{\text{Kadp}_{(\text{CDC19})}} + \frac{[\text{ATP}]}{\text{Katp}_{(\text{CDC19})}} \right)}$$

$$v_{\text{ENO1}} = \frac{[\text{ENO1}] \cdot \text{kcat}_{(\text{ENO1})} \cdot \left(\frac{[\text{2-phosphoglycerate}]}{\text{Kp2g}_{(\text{ENO1})}} - \frac{[\text{phosphoenolpyruvate}]}{\text{Kp2g}_{(\text{ENO1})} \cdot \text{Keq\_ENO}} \right)}{1 + \frac{[\text{2-phosphoglycerate}]}{\text{Kp2g}_{(\text{ENO1})}} + \frac{[\text{phosphoenolpyruvate}]}{\text{Kpep}_{(\text{ENO1})}}}$$

$$v_{\text{ENO2}} = \frac{[\text{ENO2}] \cdot \text{kcat}_{(\text{ENO2})} \cdot \left(\frac{[\text{2-phosphoglycerate}]}{\text{Kp2g}_{(\text{ENO2})}} - \frac{[\text{phosphoenolpyruvate}]}{\text{Kp2g}_{(\text{ENO2})} \cdot \text{Keq\_ENO}} \right)}{1 + \frac{[\text{2-phosphoglycerate}]}{\text{Kp2g}_{(\text{ENO2})}} + \frac{[\text{phosphoenolpyruvate}]}{\text{Kpep}_{(\text{ENO2})}}}$$

$$v_{\text{FBA}} = \frac{[\text{FBA1}] \cdot \text{kcat}_{(\text{FBA})} \cdot \left(\frac{[\text{fructose 1,6-bisphosphate}]}{\text{Kf16bp}_{(\text{FBA})}} - \frac{[\text{dihydroxyacetone phosphate}] \cdot [\text{glyceraldehyde 3-phosphate}]}{\text{Kf16bp}_{(\text{FBA})} \cdot \text{Keq}_{(\text{FBA})}} \right)}{1 + \frac{[\text{fructose 1,6-bisphosphate}]}{\text{Kf16bp}_{(\text{FBA})}} + \frac{[\text{dihydroxyacetone phosphate}]}{\text{Kdhap}_{(\text{FBA})}} + \frac{[\text{glyceraldehyde 3-phosphate}]}{\text{Kgap}_{(\text{FBA})}} + \frac{[\text{fructose 1,6-bisphosphate}] \cdot [\text{glyceraldehyde 3-phosphate}]}{\text{Kf16bp}_{(\text{FBA})} \cdot \text{Kigap}_{(\text{FBA})}} + \frac{[\text{dihydroxyacetone phosphate}] \cdot \ ,[\text{glyceraldehyde 3-phosphate}]}{\text{Kdhap}_{(\text{FBA})} \cdot \text{Kgap}_{(\text{FBA})}}}$$

$$v_{\text{GLK1}} = \frac{[\text{GLK1}] \cdot \text{kcat}_{(\text{GLK1})} \cdot \left(\frac{[\text{glucosecell}] \cdot [\text{ATP}]}{\text{Kglc}_{(\text{GLK1})} \cdot \text{Katp}_{(\text{GLK1})}} - \frac{[\text{glucose 6-phosphate}] \cdot [\text{ADP}]}{\text{Kglc}_{(\text{GLK1})} \cdot \text{Katp}_{(\text{GLK1})} \cdot \text{Keq\_HXK}} \right)}{\left(1 + \frac{[\text{glucosecell}]}{\text{Kglc}_{(\text{GLK1})}} + \frac{[\text{glucose 6-phosphate}]}{\text{Kg6p}_{(\text{GLK1})}} \right) \cdot \left(1 + \frac{[\text{ATP}]}{\text{Katp}_{(\text{GLK1})}} + \frac{[\text{ADP}]}{\text{Kadp}_{(\text{GLK1})}} \right)}$$

$$v_{\text{Glycerol3P}} = \frac{\frac{\text{Vmax}_{(\text{Glycerol3P})} \cdot [\text{glycerol 3-phosphate}]}{\text{Kg3p}_{(\text{Glycerol3P})}}}{1 + \frac{[\text{glycerol 3-phosphate}]}{\text{Kg3p}_{(\text{Glycerol3P})}}}$$

$$v_{\text{Glycerol3PDH}} = \frac{\frac{\text{Vmax}_{(\text{Glycerol3PDH})}}{\text{Kdhap}_{(\text{Glycerol3PDH})} \cdot \text{Knadh}_{(\text{Glycerol3PDH})}} \cdot \left([\text{dihydroxyacetone phosphate}] \cdot [\text{NADH}] - \frac{[\text{glycerol 3-phosphate}] \cdot [\text{NAD}]}{\text{Keq}_{(\text{Glycerol3PDH})}} \right)}{\left(1 + \frac{[\text{fructose 1,6-bisphosphate}]}{\text{Kfbp}_{(\text{Glycerol3PDH})}} + \frac{[\text{ATP}]}{\text{Katp}_{(\text{Glycerol3PDH})}} + \frac{[\text{ADP}]}{\text{Kadp}_{(\text{Glycerol3PDH})}} \right) \cdot \left(1 + \frac{[\text{dihydroxyacetone phosphate}]}{\text{Kdhap}_{(\text{Glycerol3PDH})}} + \frac{[\text{glycerol ;3-phosphate}]}{\text{Kg3p}_{(\text{Glycerol3PDH})}} \right) \cdot \left(1 + \frac{[\text{NADH}]}{\text{Knadh}_{(\text{Glycerol3PDH})}} + \frac{[\text{NAD}]}{\text{Knad}_{(\text{Glycerol3PDH})}} \right)}$$

$$v_{\text{GPM1}} = \frac{[\text{GPM1}] \cdot \text{kcat}_{(\text{GPM1})} \cdot \left(\frac{[\text{3-phosphoglycerate}]}{\text{Kp3g}_{(\text{GPM1})}} - \frac{[\text{2-phosphoglycerate}]}{\text{Kp3g}_{(\text{GPM1})} \cdot \text{Keq}_{(\text{GPM1})}} \right)}{1 + \frac{[\text{3-phosphoglycerate}]}{\text{Kp3g}_{(\text{GPM1})}} + \frac{[\text{2-phosphoglycerate}]}{\text{Kp2g}_{(\text{GPM1})}}}$$

$$v_{\text{HXK1}} = \frac{[\text{HXK1}] \cdot \text{kcat}_{(\text{HXK1})} \cdot \left(\frac{[\text{glucose\_cell}] \cdot [\text{ATP}]}{\text{Kglc}_{(\text{HXK1})} \cdot \text{Katp}_{(\text{HXK1})}} - \frac{[\text{glucose 6-phosphate}] \cdot [\text{ADP}]}{\text{Kglc}_{(\text{HXK1})} \cdot \text{Katp}_{(\text{HXK1})} \cdot \text{Keq\_HXK}} \right)}{\left(1 + \frac{[\text{glucose\_cell}]}{\text{Kglc}_{(\text{HXK1})}} + \frac{[\text{glucose 6-phosphate}]}{\text{Kg6p}_{(\text{HXK1})}} + \frac{[\text{trehalose 6-phosphate}]}{\text{Kit6p}_{(\text{HXK1})}} \right) \cdot \left(1 + \frac{[\text{ATP}]}{\text{Katp}_{(\text{HXK1})}} + \frac{[\text{ADP}]}{\text{Kadp}_{(\text{HXK1})}} \right)}$$

$$v_{\text{HXK2}} = \frac{[\text{HXK2}] \cdot \text{kcat}_{(\text{HXK2})} \cdot \left(\frac{[\text{glucose\_cell}] \cdot [\text{ATP}]}{\text{Kglc}_{(\text{HXK2})} \cdot \text{Katp}_{(\text{HXK2})}} - \frac{[\text{glucose 6-phosphate}] \cdot [\text{ADP}]}{\text{Kglc}_{(\text{HXK2})} \cdot \text{Katp}_{(\text{HXK2})} \cdot \text{Keq\_HXK}} \right)}{\left(1 + \frac{[\text{glucose\_cell}]}{\text{Kglc}_{(\text{HXK2})}} + \frac{[\text{glucose 6-phosphate}]}{\text{Kg6p}_{(\text{HXK2})}} + \frac{[\text{trehalose 6-phosphate}]}{\text{Kit6p}_{(\text{HXK2})}} \right) \cdot \left(1 + \frac{[\text{ATP}]}{\text{Katp}_{(\text{HXK2})}} + \frac{[\text{ADP}]}{\text{Kadp}_{(\text{HXK2})}} \right)}$$

$$v_{\text{HXT}} = \frac{\frac{\text{Vmax}_{(\text{HXT})} \cdot ([\text{glucoseextracellular}] - [\text{glucosecell}])}{\text{Kglc}_{(\text{HXT})}}}{1 + \frac{[\text{glucoseextracellular}]}{\text{Kglc}_{(\text{HXT})}} + \frac{[\text{glucosecell}]}{\text{Kglc}_{(\text{HXT})}} + \frac{\frac{\text{Ki}_{(\text{HXT})} \cdot [\text{glucoseextracellular}]}{\text{Kglc}_{(\text{HXT})}} \cdot [\text{glucosecell}]}{\text{Kglc}_{(\text{HXT})}}}$$

$$v_{\text{PDC1}} = \frac{\frac{[\text{PDC1}] \cdot \text{kcat}_{(\text{PDC1})} \cdot [\text{pyruvate}]}{\text{Kpyr}_{(\text{PDC1})}}}{1 + \frac{[\text{pyruvate}]}{\text{Kpyr}_{(\text{PDC1})}}}$$

$$v_{\text{PDC5}} = \frac{\frac{[\text{PDC5}] \cdot \text{kcat}_{(\text{PDC5})} \cdot [\text{pyruvate}]}{\text{Kpyr}_{(\text{PDC5})}}}{1 + \frac{[\text{pyruvate}]}{\text{Kpyr}_{(\text{PDC5})}}}$$

$$v_{\text{PDC6}} = \frac{\frac{[\text{PDC6}] \cdot \text{kcat}_{(\text{PDC6})} \cdot [\text{pyruvate}]}{\text{Kpyr}_{(\text{PDC6})}}}{1 + \frac{[\text{pyruvate}]}{\text{Kpyr}_{(\text{PDC6})}}}$$

$$v_{\text{PFK}} = \min([\text{PFK1}],[\text{PFK2}]) \cdot \text{kcat}_{(\text{PFK})} \cdot \frac{\text{gR}_{(\text{PFK})} \cdot \frac{[\text{fructose 6-phosphate}]}{\text{Kf6p}_{(\text{PFK})}} \cdot \frac{[\text{ATP}]}{\text{Katp}_{(\text{PFK})}} \cdot \left(1 - \frac{\frac{[\text{fructose 1,6-bisphosphate}] \cdot [\text{ADP}]}{[\text{fructose 6-phosphate}] \cdot [\text{ATP}]}}{\text{Keq}_{(\text{PFK})}} \right) \cdot \left(1 + \frac{[\text{fructose 6-phosphate}]}{\text{Kf6p}_{(\text{PFK})}} + \frac{[\text{ATP}]}{\text{Katp}_{(\text{PFK})}} + \frac{\frac{\text{gR}_{(\text{PFK})} \cdot [\text{fructose 6-phosphate}]}{\text{Kf6p}_{(\text{PFK})}} \cdot [\text{ATP}]}{\text{Katp}\ \ mathrm(PFK)} + \frac{[\text{fructose 1,6-bisphosphate}]}{\text{Kf16}_{(\text{PFK})}} + \frac{[\text{ADP}]}{\text{Kadp}_{(\text{PFK})}} + \frac{\frac{\text{gR}_{(\text{PFK})} \cdot [\text{fructose 1,6-bisphosphate}]}{\text{Kf16}_{(\text{PFK})}} \cdot [\text{ADP}]}{\text{Kadp}_{(\text{PFK})}} \right)}{\left(1 + \frac{[\text{fructose 6-phosphate}]}{\text{Kf6p}_{(\text{PFK})}} + \frac{[\text{ATP}]}{\text{Katp}_{(\text{PFK})}} + \frac{\frac{\text{gR}_{(\text{PFK})} \cdot [\text{fructose 6-phosphate}]}{\text{Kf6p}_{(\text{PFK})}} \cdot [\text{ATP}]}{\text{Katp}_{(\text{PFK})}} + \frac{[\text{fructose 1,6-bisphosphate}]}{\text{Kf16}_{(\text{PFK})}} + \frac{[\text{ADP}]}{\text{Kadp}_{(\text{PFK})}} + \frac{\frac{\text{gR}_{(\text{PFK})} \cdot \ \ mathrm[fructose\ 1,6-bisphosphate]}{\text{Kf16}_{(\text{PFK})}} \cdot [\text{ADP}]}{\text{Kadp}_{(\text{PFK})}} \right)^2 + \text{L0}_{(\text{PFK})} \cdot \left(\frac{1 + \frac{\text{Ciatp}_{(\text{PFK})} \cdot [\text{ATP}]}{\text{Kiatp}_{(\text{PFK})}}}{1 + \frac{[\text{ATP}]}{\text{Kiatp}_{(\text{PFK})}}} \right)^2 \cdot \left(\frac{1 + \frac{\text{Camp}_{(\text{PFK})} \cdot [\text{AMP}]}{\text{Kamp}_{(\text{PFK})}}}{1 + \frac{[\text{AMP}]}{\text{Kamp}_{(\text{PFK})}}} \right)^2 \cdot \left(\frac{1 + \frac{\text{Cf26}_{(\text{PFK})} \cdot [\text{fructose 2,6-bisphosphate}]}{\text{Kf26}_{(\text{PFK})}} + \frac{\text{Cf16}_{(\text{PFK})} \cdot [\text{fructose 1,6-bisphosphate}]}{\text{Kf16}_{(\text{PFK})}}}{1 + \frac{[\text{fructose 2,6-bisphosphate}]}{\text{Kf26}_{(\text{PFK})}} + \frac{[\text{fructose 1,6-bisphosphate}]}{\text{Kf16}_{(\text{PFK})}}} \right)^2 \cdot \left(1 + \frac{\text{Catp}_{(\text{PFK})} \cdot [\text{ATP}]}{\text{Katp}_{(\text{PFK})}} \right)^2}$$

$$v_{\text{PGI}} = \frac{[\text{PGI1}] \cdot \text{kcat}_{(\text{PGI})} \cdot \left(\frac{[\text{glucose 6-phosphate}]}{\text{Kg6p}_{(\text{PGI})}} - \frac{[\text{fructose 6-phosphate}]}{\text{Kg6p}_{(\text{PGI})} \cdot \text{Keq}_{(\text{PGI})}} \right)}{1 + \frac{[\text{glucose 6-phosphate}]}{\text{Kg6p}_{(\text{PGI})}} + \frac{[\text{fructose 6-phosphate}]}{\text{Kf6p}_{(\text{PGI})}}}$$

$$v_{\text{PGK1}} = \frac{[\text{PGK1}] \cdot \text{kcat}_{(\text{PGK1})} \cdot \left(\frac{[\text{ADP}]}{\text{Kadp}_{(\text{PGK1})}} \right)^{\text{nHadp}_{(\text{PGK1})} - 1} \cdot \left(\frac{[\text{1,3-bisphosphoglycerate}] \cdot [\text{ADP}]}{\text{Kbpg}_{(\text{PGK1})} \cdot \text{Kadp}_{(\text{PGK1})}} - \frac{[\text{3-phosphoglycerate}] \cdot [\text{ATP}]}{\text{Kbpg}_{(\text{PGK1})} \cdot \text{Kadp}_{(\text{PGK1})} \cdot \text{Keq}_{(\text{PGK1})}} \right)}{\left(1 + \frac{[\text{1,3-bisphosphoglycerate}]}{\text{Kbpg}_{(\text{PGK1})}} + \frac{[\text{3-phosphoglycerate}]}{\text{Kp3g}_{(\text{PGK1})}} \right) \cdot \left(1 + \left(\frac{[\text{ADP}]}{\text{Kadp}_{(\text{PGK1})}} \right)^{\text{nHadp}_{(\text{PGK1})}} + \frac{[\text{ATP}]}{\text{Katp}_{(\text{PGK1})}} \right)}$$

$$v_{\text{PGM}} = \frac{\text{Vmax}_{(\text{PGM})} \cdot \left(\frac{[\text{glucose 6-phosphate}]}{\text{Kg6p}_{(\text{PGM})}} - \frac{[\text{glucose 1-phosphate}]}{\text{Kg6p}_{(\text{PGM})} \cdot \text{Keq}_{(\text{PGM})}} \right)}{1 + \frac{[\text{glucose 6-phosphate}]}{\text{Kg6p}_{(\text{PGM})}} + \frac{[\text{glucose 1-phosphate}]}{\text{Kg1p}_{(\text{PGM})}}}$$

$$v_{\text{PYK2}} = \frac{\frac{[\text{PYK2}] \cdot \text{kcat}_{(\text{PYK2})} \cdot \left([\text{phosphoenolpyruvate}] \cdot [\text{ADP}] - \frac{[\text{pyruvate}] \cdot [\text{ATP}]}{\text{Keq\_PYK}} \right)}{\text{Kpep}_{(\text{PYK2})} \cdot \text{Kadp}_{(\text{PYK2})}}}{\left(1 + \frac{[\text{phosphoenolpyruvate}]}{\text{Kpep}_{(\text{PYK2})}} + \frac{[\text{pyruvate}]}{\text{Kpyr}_{(\text{PYK2})}} + \text{L0}_{(\text{PYK2})} \cdot \frac{\frac{[\text{ATP}]}{\text{Kiatp}_{(\text{PYK2})}} + 1}{\frac{[\text{fructose 1,6-bisphosphate}]}{\text{Kf16p}_{(\text{PYK2})}} + 1} \right) \cdot \left(1 + \frac{[\text{ADP}]}{\text{Kadp}_{(\text{PYK2})}} + \frac{[\text{ATP}]}{\text{Katp}_{(\text{PYK2})}} \right)}$$

$$v_{\text{succinate branch}} = k_{(\text{succinate branch})} \cdot [\text{pyruvate}] \cdot [\text{NAD}]$$

$$v_{\text{T6P synthase}} = \frac{\frac{\text{Vmax}_{(\text{T6P synthase})} \cdot [\text{glucose 6}-\text{phosphate}] \cdot [\text{UDP glucose}]}{\text{Kg6p}_{(\text{T6P synthase})} \cdot \text{Kudg}_{(\text{T6P synthase})}}}{\left(1 + \frac{[\text{glucose 6}-\text{phosphate}]}{\text{Kg6p}_{(\text{T6P synthase})}}\right) \cdot \left(1 + \frac{[\text{UDP glucose}]}{\text{Kudg}_{(\text{T6P synthase})}}\right)}$$

$$v_{\text{TDH1}} = \frac{[\text{TDH1}] \cdot \text{kcat}_{(\text{TDH1})} \cdot \left(\frac{[\text{glyceraldehyde 3}-\text{phosphate}] \cdot [\text{NAD}]}{\text{Kgap}_{(\text{TDH1})} \cdot \text{Knad}_{(\text{TDH1})}} - \frac{[\text{1,3}-\text{bisphosphoglycerate}] \cdot [\text{NADH}]}{\text{Kgap}_{(\text{TDH1})} \cdot \text{Knad}_{(\text{TDH1})} \cdot \text{Keq\_TDH}}\right)}{\left(1 + \frac{[\text{glyceraldehyde 3}-\text{phosphate}]}{\text{Kgap}_{(\text{TDH1})}} + \frac{[\text{1,3}-\text{bisphosphoglycerate}]}{\text{Kbpg}_{(\text{TDH1})}}\right) \cdot \left(1 + \frac{[\text{NAD}]}{\text{Knad}_{(\text{TDH1})}} + \frac{[\text{NADH}]}{\text{Knadh}_{(\text{TDH1})}}\right)}$$

$$v_{\text{TDH2}} = \frac{[\text{TDH2}] \cdot \text{kcat}_{(\text{TDH2})} \cdot \left(\frac{[\text{glyceraldehyde 3}-\text{phosphate}] \cdot [\text{NAD}]}{\text{Kgap}_{(\text{TDH2})} \cdot \text{Knad}_{(\text{TDH2})}} - \frac{[\text{1,3}-\text{bisphosphoglycerate}] \cdot [\text{NADH}]}{\text{Kgap}_{(\text{TDH2})} \cdot \text{Knad}_{(\text{TDH2})} \cdot \text{Keq\_TDH}}\right)}{\left(1 + \frac{[\text{glyceraldehyde 3}-\text{phosphate}]}{\text{Kgap}_{(\text{TDH2})}} + \frac{[\text{1,3}-\text{bisphosphoglycerate}]}{\text{Kbpg}_{(\text{TDH2})}}\right) \cdot \left(1 + \frac{[\text{NAD}]}{\text{Knad}_{(\text{TDH2})}} + \frac{[\text{NADH}]}{\text{Knadh}_{(\text{TDH2})}}\right)}$$

$$v_{\text{TDH3}} = \frac{[\text{TDH3}] \cdot \text{kcat}_{(\text{TDH3})} \cdot \left(\frac{[\text{glyceraldehyde 3}-\text{phosphate}] \cdot [\text{NAD}]}{\text{Kgap}_{(\text{TDH3})} \cdot \text{Knad}_{(\text{TDH3})}} - \frac{[\text{1,3}-\text{bisphosphoglycerate}] \cdot [\text{NADH}]}{\text{Kgap}_{(\text{TDH3})} \cdot \text{Knad}_{(\text{TDH3})} \cdot \text{Keq\_TDH}}\right)}{\left(1 + \frac{[\text{glyceraldehyde 3}-\text{phosphate}]}{\text{Kgap}_{(\text{TDH3})}} + \frac{[\text{1,3}-\text{bisphosphoglycerate}]}{\text{Kbpg}_{(\text{TDH3})}}\right) \cdot \left(1 + \frac{[\text{NAD}]}{\text{Knad}_{(\text{TDH3})}} + \frac{[\text{NADH}]}{\text{Knadh}_{(\text{TDH3})}}\right)}$$

$$v_{\text{TPI}} = \frac{\frac{[\text{TPI1}] \cdot \text{kcat}_{(\text{TPI})}}{\text{Kdhap}_{(\text{TPI})}} \cdot \left([\text{dihydroxyacetone phosphate}] - \frac{[\text{glyceraldehyde 3}-\text{phosphate}]}{\text{Keq}_{(\text{TPI})}}\right)}{1 + \frac{[\text{dihydroxyacetone phosphate}]}{\text{Kdhap}_{(\text{TPI})}} + \frac{[\text{glyceraldehyde 3}-\text{phosphate}]}{\text{Kgap}_{(\text{TPI})}} \cdot \left(1 + \left(\frac{[\text{glyceraldehyde 3}-\text{phosphate}]}{\text{Kigap}_{(\text{TPI})}}\right)^4\right)}$$

$$v_{\text{UDP}-\text{glucose phosphorylase}} = \frac{\frac{\text{Vmax}_{(\text{UDP}-\text{glucose phosphorylase})} \cdot [\text{UTP}] \cdot [\text{glucose 1}-\text{phosphate}]}{\text{Kutp}_{(\text{UDP}-\text{glucose phosphorylase})} \cdot \text{Kg1p}_{(\text{UDP}-\text{glucose phosphorylase})}}}{\frac{\text{Kiutp}_{(\text{UDP}-\text{glucose phosphorylase})}}{\text{Kutp}_{(\text{UDP}-\text{glucose phosphorylase})}} + \frac{[\text{UTP}]}{\text{Kutp}_{(\text{UDP}-\text{glucose phosphorylase})}} + \frac{[\text{glucose 1}-\text{phosphate}]}{\text{Kg1p}_{(\text{UDP}-\text{glucose phosphorylase})}} + \frac{[\text{UTP}] \cdot [\text{glucose 1}-\text{phosphate}]}{\text{Kutp}_{(\text{UDP}-\text{glucose phosphorylase})} \cdot \text{Kg1p}_{(\text{UDP}-\text{glucose phosphorylase})}} + \frac{\frac{\text{Kiutp}_{(\text{UDP}-\text{glucose phosphorylase})}}{\text{Kutp}_{(\text{UDP}-\text{glucose phosphorylase})}} \cdot [\text{UDP glucose}]}{\text{Kiudg}_{(\text{UDP}-\text{glucose phosphorylase})}} + \frac{[\text{glucose 1}-\text{phosphate}] \cdot [\text{UDP glucose}]}{\text{Kg1p}_{(\text{UDP}-\text{glucose phosphorylase})} \cdot \text{Kiudg}_{(\text{UDP}-\text{glucose phosphorylase})}}}$$

$$v_{\text{UDP to UTP pseudoreaction}} = k_{(\text{UDP to UTP pseudoreaction})} \cdot [\text{UDP}] \cdot [\text{ATP}]$$

Differential equations

$$\frac{d([\text{ADP}])}{dt} = +v_{\text{UDP to UTP pseudoreaction}} - v_{\text{PYK2}} - v_{\text{CDC19}} - v_{\text{PGK1}} + v_{\text{PFK}} + v_{\text{HXK2}} + v_{\text{HXK1}} + v_{\text{GLK1}} + v_{\text{ATPase}} - 2 \cdot v_{\text{AK}}$$

$$\frac{d([\text{ATP}])}{dt} = -v_{\text{UDP to UTP pseudoreaction}} + v_{\text{PYK2}} + v_{\text{CDC19}} + v_{\text{PGK1}} - v_{\text{PFK}} - v_{\text{HXK2}} - v_{\text{HXK1}} - v_{\text{GLK1}} - v_{\text{ATPase}} + v_{\text{AK}}$$

$$\frac{d([\text{acetaldehyde}])}{dt} = +v_{\text{PDC6}} + v_{\text{PDC5}} + v_{\text{PDC1}} - v_{\text{acetate branch}} - v_{\text{ADH5}} - v_{\text{ADH1}}$$

$$\frac{d([\text{1,3}-\text{bisphosphoglycerate}])}{dt} = +v_{\text{TDH1}} - v_{\text{PGK1}} + v_{\text{TDH2}} + v_{\text{TDH3}}$$

$$\frac{d([\text{dihydroxyacetone phosphate}])}{dt} = +v_{\text{FBA}} - v_{\text{TPI}} - v_{\text{Glycerol3PDH}}$$

$$\frac{d([\text{fructose 1,6}-\text{bisphosphate}])}{dt} = +v_{\text{PFK}} - v_{\text{FBA}}$$

$$\frac{d([\text{fructose 6}-\text{phosphate}])}{dt} = +v_{\text{PGI}} - v_{\text{PFK}}$$

$$\frac{d([\text{glucose 1}-\text{phosphate}])}{dt} = +v_{\text{PGM}} - v_{\text{UDP-glucose phosphorylase}}$$

$$\frac{d([\text{glycerol 3}-\text{phosphate}])}{dt} = -v_{\text{Glycerol3P}} + v_{\text{Glycerol3PDH}}$$

$$\frac{d([\text{glucose 6}-\text{phosphate}])}{dt} = -v_{\text{PGM}} - v_{\text{PGI}} + v_{\text{HXK2}} + v_{\text{HXK1}} + v_{\text{GLK1}} - v_{\text{T6Psynth}}$$

$$\frac{d([\text{glyceraldehyde 3}-\text{phosphate}])}{dt} = -v_{\text{TDH1}} + v_{\text{FBA}} + v_{\text{TPI}} - v_{\text{TDH3}} - v_{\text{TDH2}}$$

$$\frac{d([\text{glucose\_cell}])}{dt} = +v_{\text{HXT}} - v_{\text{HXK2}} - v_{\text{HXK1}} - v_{\text{GLK1}}$$

$$\frac{d([\text{NAD}])}{dt} = -v_{\text{TDH1}} - 3 \cdot v_{\text{succinate branch}} + v_{\text{Glycerol3PDH}} - v_{\text{acetate branch}} + v_{\text{ADH5}} + v_{\text{ADH1}} - v_{\text{TDH3}} - v_{\text{TDH2}}$$

$$\frac{d([\text{2}-\text{phosphoglycerate}])}{dt} = +v_{\text{GPM1}} - v_{\text{ENO1}} - v_{\text{ENO2}}$$

$$\frac{d([\text{3}-\text{phosphoglycerate}])}{dt} = +v_{\text{PGK1}} - v_{\text{GPM1}}$$

$$\frac{d([\text{phosphoenolpyruvate}])}{dt} = -v_{\text{PYK2}} - v_{\text{CDC19}} + v_{\text{ENO1}} + v_{\text{ENO2}}$$

$$\frac{d([\text{pyruvate}])}{dt} = +v_{\text{PYK2}} + v_{\text{CDC19}} - v_{\text{PDC6}} - v_{\text{PDC5}} - v_{\text{PDC1}} - v_{\text{succinate branch}}$$

$$\frac{d([\text{trehalose 6}-\text{phosphate}])}{dt} = +v_{\text{T6Psynth}} - v_{\text{T6P phosphatase}}$$

$$\frac{d([\text{UDP}])}{dt} = -v_{\text{UDP to UTP pseudoreaction}} + v_{\text{T6Psynth}}$$

$$\frac{d([\text{UTP}])}{dt} = +v_{\text{UDP to UTP pseudoreaction}} - v_{\text{UDP-glucose phosphorylase}}$$

Mass conservation

$$[\text{AMP}] = \text{sum\_AXP} - [\text{ATP}] - [\text{ADP}]$$

$$[\text{NADH}] = \text{sum\_NAD} - [\text{NAD}]$$

$$[\text{UDP-glucose}] = \text{sum\_UXP} - [\text{UTP}] - [\text{UDP}]$$

$$\text{energy\_charge} = \frac{[\text{ATP}] + \frac{[\text{ADP}]}{2}}{\text{sum\_AXP}}$$
